# PD-L2 negatively regulates Th1-mediated immunopathology during *Fasciola hepatica* infection

**DOI:** 10.18632/oncotarget.12790

**Published:** 2016-10-21

**Authors:** Cinthia C. Stempin, Claudia C. Motrán, María P. Aoki, Cristian R. Falcón, Fabio M. Cerbán, Laura Cervi

**Affiliations:** ^1^ Centro de Investigaciones en Bioquímica Clínica e Inmunología (CIBICI), CONICET, Departamento de Bioquímica Clínica, Facultad de Ciencias Químicas, Universidad Nacional de Córdoba, Medina Allende y Haya de la Torre, Ciudad Universitaria, Córdoba, Argentina

**Keywords:** macrophage, PD-L2, F. hepatica, Th1 response, infection

## Abstract

Macrophage plasticity is critical for controlling inflammation including those produced by helminth infections, where alternatively activated macrophages (AAM) are accumulated in tissues. AAM expressing the co-inhibitory molecule programmed death ligand 2 (PD-L2), which is capable of binding programmed death 1 (PD-1) expressed on activated T cells, have been demonstrated in different parasitic infections. However, the role of PD-L2 during *F. hepatica* infection has not yet been explored. We observed that *F. hepatica* infection or a *F. hepatica* total extract (TE) injection increased the expression of PD-L2 on peritoneal macrophages. In addition, the absence of PD-L2 expression correlated with an increase in susceptibility to *F. hepatica* infection, as evidenced by the shorter survival and increased liver damage observed in PD-L2 deficient (KO) mice. We assessed the contribution of the PD-L2 pathway to Th2 polarization during this infection, and found that the absence of PD-L2 caused a diminished Th2 type cytokine production by TE stimulated splenocytes from PD-L2 KO infected compared with WT mice. Besides, splenocytes and intrahepatic leukocytes from infected PD-L2 KO mice showed higher levels of IFN-γ than those from WT mice. Arginase expression and activity and IL-10 production were reduced in macrophages from PD-L2 KO mice compared to those from WT mice, revealing a strong correlation between PD-L2 expression and AAM polarization. Taken together, our data indicate that PD-L2 expression in macrophages is critical for AAM induction and the maintenance of an optimal balance between the Th1- and Th2-type immune responses to assure host survival during *F. hepatica* infection.

## INTRODUCTION

The helminth parasite *F. hepatica* is the causative agent of liver fluke disease (fasciolosis) in cattle and sheep, with the liver damage caused by these parasites reducing animal performance, fertility, wool and milk production, and resulting in huge economic losses worldwide [1]. Moreover, fasciolosis is becoming an emerging disease in humans, with a high prevalence in some regions of the planet such as Bolivian Altiplano [2].

The parasite's life cycle involves a complex interplay between parasite and host. Following ingestion by grazing animals, the parasites emerge from their cysts in the intestine, traverse the intestinal wall, in just a few hours, before migrating through the liver capsule and into the parenchyma. There, their feeding and migratory activities cause tissue perforation and hemorrhage, leading to extensive tissue damage. After about 7–8 weeks, the parasites migrate into the bile ducts, mature and produce eggs which are released onto the pasture with the faeces [3]. It is assumed that during the evolution both, the host's immune system and the parasites, exert reciprocal selective pressures on each other which leads to rapid reciprocal adaptation to survival. Thus, *F. hepatica* infection in mice induces a strong Th2 immune response with high IL-4, IL-5 and IL-10 but little IFN-γ production by parasite stimulated splenocytes [4-6]. In the same way, although in infected cattle both Th1- and Th2-type immune responses are present at the early stages of infection, a high prevalence of the Th2 response is observed when the infection is established [7]. In addition, *F. hepatica* infection as well as the excretory-secretory products (ES) released by the parasite are immunomodulatory, promoting Treg induction and AAM that suppress the Th1-type immune and promote Th2 development through IL-10 production [8-11]. Collectively, this prevents the development of a protective Th1-type immune response against the parasite, thereby allowing the development of a chronic infection.

The immunomodulatory capacity of *F. hepatica* products has been shown in murine models of co-infection with other pathogens such as *Bordetella pertussis*, in which the cellular immune response against *Bordetella* is abrogated resulting in a delayed clearance of the bacteria and poor *B. pertussis*-specific IFN-γ production [4]. Furthermore, injection of purified cathepsin L proteinase from *F. hepatica* is capable of suppressing *B. pertussis*-specific IFN-γ production by a IL-4-dependent mechanism [12]. In line with this, a correlation between increased levels of IL-4 with decreased IFN-γ production was observed in calves co-infected with *F. hepatica* and *Mycobacterium bovis* BCG [13], thus highlighting the suppressive effect of *F. hepatica* on the IFN-γ production induced by a concurrent pathogen.

T cell activation and tolerance are controlled by costimulatory molecules with the PD-1 pathway involving the negative signaling of the accessory molecules PD-L1 and PD-L2, which bind PD-1 expressed in activated T cells inhibiting their proliferation [14, 15]. In particular, PD-L2 is a negative regulator of T cell activation, but is also essential for the regulation of T cell tolerance [16]. During helminth infections, the suppression of T cell responses by PD-1 has been mostly attributed to macrophages expressing PD-L1 and /or PD-L2, and the PD-1 pathway has been shown to be an important mechanism of suppression by AAM [17, 18]. Related to this, it has recently been demonstrated that *F. hepatica* ES induce CD4+ T cell anergy via selective up-regulation of PD-L2 expression in macrophages [19]. Furthermore, blocking of PD-L2 in co-cultures of anti-CD3-stimulated CD4+ T cells with ES-treated macrophages increases IFN-γ and decreases IL-10 production [19]. From these results, it appears that ES from *F. hepatica* might exert a control of the Th1-type response through PD-L2.

Based on the above findings, and given that protective immune response against *F. hepatica* is mostly related to the induction of Th1 responses, we investigated the role of PD-L2 expression during *F. hepatica* infection and its influence on the control of the protective immune response and the immunopathology. Here, we demonstrated that PD-L2 expression was up-regulated in macrophages during *F. hepatica* infection, and that its expression was critical for inducing AAM. In addition, the absence of PD-L2 expression was correlated with an increase susceptibility to *F. hepatica* infection, as evidenced by a reduction in survival rate and increased liver damage in PD-L2 KO mice. Concomitantly, a reduction in the production of the Th2-type cytokines IL-4 and IL-10 was observed in splenocytes from infected PD-L2 KO mice, and an increased production of IFN-γ was found in splenocytes and intrahepatic leucocytes from these animals. Based on these observations, we postulate that increased PD-L2 expression in macrophages at early time post-infection may be involved in the control of the incipient Th1 response to protect the host from an excessive immune response that could cause liver damage.

## RESULTS

### *F. hepatica* infection increased PD-L2 expression in peritoneal macrophages

**Figure 1:**
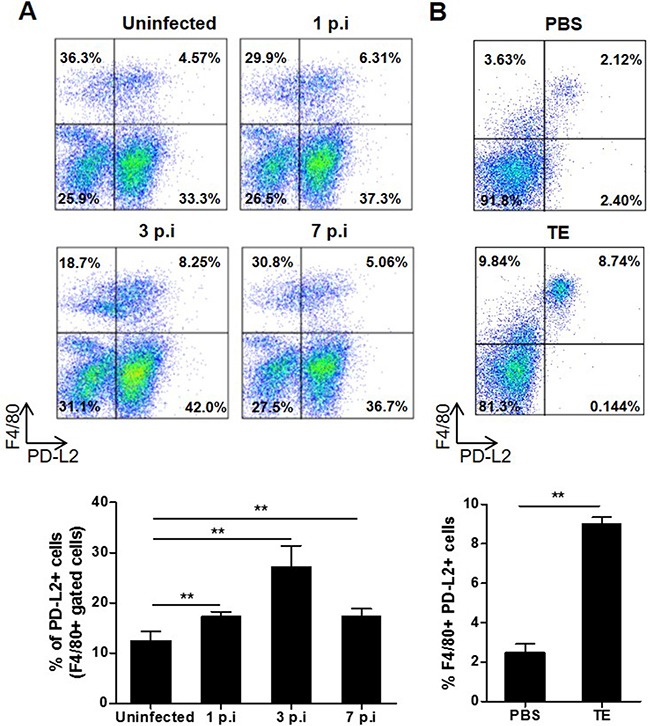
Effect of *F. hepatica* infection or total extract antigen injection on PD-L2 expression by peritoneal macrophages **A.** BALB/c mice were infected with 8 *F. hepatica* metacercariae, and PC were isolated at days 1, 3 and 7 p.i. Uninfected animals were analyzed in parallel as controls. PC were processed by flow cytometry and analyzed for F4/80+PD-L2+ cells. Representative dot plots of each group of mice are shown, with bars displaying the percentage of PD-L2+ cells gated on F4/80+ cells, ** *p<* 0.005. **B.** BALB/c WT mice were injected with 80 μg of TE or PBS as control and the PC were obtained 24 h later. F4/80+ PD-L2+ cells were analyzed by flow cytometry. A representative dot plot for each group of mice is shown. Bars display percentage of F4/80+ PD-L2+ cells, **p=0.0038. Data are representative of two independent experiments and expressed as mean ± SD.

During helminth infections, there is a predominant Th2 response characterized by a high production of IL-4, IL-5, IL-9, IL-10 and IL-13 cytokines [20]. In addition, it has been shown that the costimulatory molecule PD-L2 expressed in macrophages, among other cells, is strongly induced by IL-4 and correlates with other established markers for AAM, thereby serving as a useful molecule to identify AAM [21]. *F. hepatica*, in common with other helminth parasites, induces AAM in murine and ruminants through several products such as ES, protein thioredoxin peroxidase and fatty acid binding protein [8-10]. Taking the above into account, we first studied whether *F. hepatica* infection or TE injection were able to modify PD-L2 expression in peritoneal macrophages, since the parasite migrates through the peritoneal cavity before reaching the liver. To carry this out, we evaluated at different times post infection (p.i) the percentage of F4/80^+^ PD-L2^+^ cells in the peritoneal lavage of *F. hepatica* infected and TE injected mice. We observed that in both infected (Figure 1A) and TE injected (Figure 1B) groups of mice, the percentage of macrophages that expressed PD-L2 was significantly increased compared with uninfected or PBS injected mice, respectively.

### PD-L2 expression is involved in *F. hepatica* infection outcome

**Figure 2:**
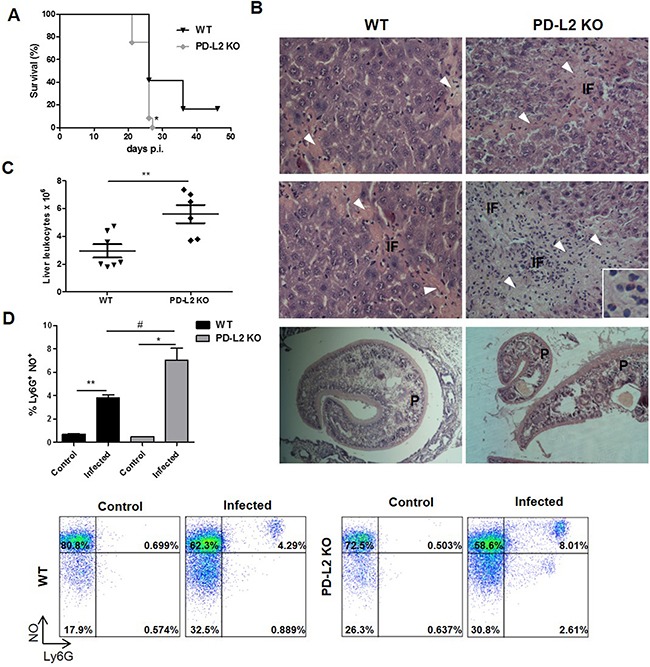
Increased susceptibility to *F. hepatica* infection in PD-L2 KO mice WT and PD-L2 KO mice were orally infected with 8 *F. hepatica* metacercariae. **A**. Survival was followed for 50 days, *p<0.02, Gehan–Breslow Wilcoxon test, n=12 per group. **B.** Representative hematoxylin and eosin images of livers from each group are shown (400x). Arrow heads in fibrosis areas, inflammatory foci (IF) and the presence of worms (P) are indicated. Inset shows the extensive inflammatory infiltrate. **C.** Intrahepatic leukocytes were isolated by percoll gradient and quantified in a Neubauer chamber. Each dot represents the total liver leukocyte number in individual livers of each group *p*=0.0065. D. Intrahepatic leukocytes were stained with anti-Ly6G before being incubated with 20μM of DAF-FMDA probe for intracellular NO detection. Bars show the percentage of Ly6G+NO+ cells. Representative dot plots of each group of control or infected WT and PD-L2 mice are shown,**p= 0.021; * *p*=0.0149; # *p*=0.0354. The data are representative of two experiments with similar results, and are expressed as mean ± SD.

To determine the relevance of PD-L2 expression during *F. hepatica* infection, susceptible BALB/c WT and PD-L2 KO mice were infected and the survival rates evaluated. After infection, almost all control mice (WT) died between days 26 to 47, while all PD-L2 KO mice died in the period 22 to 28 days p.i (Figure 2A). The level of damage was evaluated in the liver (target organ of infection) by analyzing the histopathology changes, with PD-L2 KO mice revealing a wide range of hepatic lesions and inflammatory signs (Figure 2B and Table 1). Livers from both WT and PD-L2 KO mice showed granulocytic inflammatory infiltrate, necrosis, hemorrhage and fibrosis, with all these characteristics being more severe in the livers from PD-L2 KO mice (Figure 2B and Table 1). In addition, the total liver leukocyte infiltration was significantly increased in PD-L2 KO compared to WT infected mice (Figure 2C).

**Table 1:** Histopathological changes in livers from WT and PD-L2 KO mice infected with *F. hepatica*

	WT	PD-L2 KO
**Necrosis^a^**	+++	++++
**Worms^a^**	++	++
**Inflammatory infiltrate^a^**	+++	++++
**(main cell type)^b^**	(PMN/Mo/Eo/Ly)	(PMN/Mo/Eo/Ly)
**Hemorragic foci^a^**	+	+++
**Fibrosis^a^**	+	+++

a Scale: + mild, ++ moderate; +++ severe; ++++ very severe.

b PMN: Polymorfonuclear cells; Mo: monocytes; Eo: eosinophils; Ly: lymphocytes.

In order to determine whether liver infiltrating leukocytes can produce any mediators able to favor hepatic lesion, we measured NO production by Ly6G+ cells and found that the percentage of NO-producing cells was higher in PD-L2 KO compared to WT infected mice (8,01 *vs* 4,29 respectively) (Figure 2D).

Taken together, the above results indicate that PD-L2 expression is important for prolonging the survival of *F. hepatica* infected mice by restricting inflammatory liver damage.

### PD-L2 expression is involved in Th1-Th2 balance and macrophage activation

**Figure 3:**
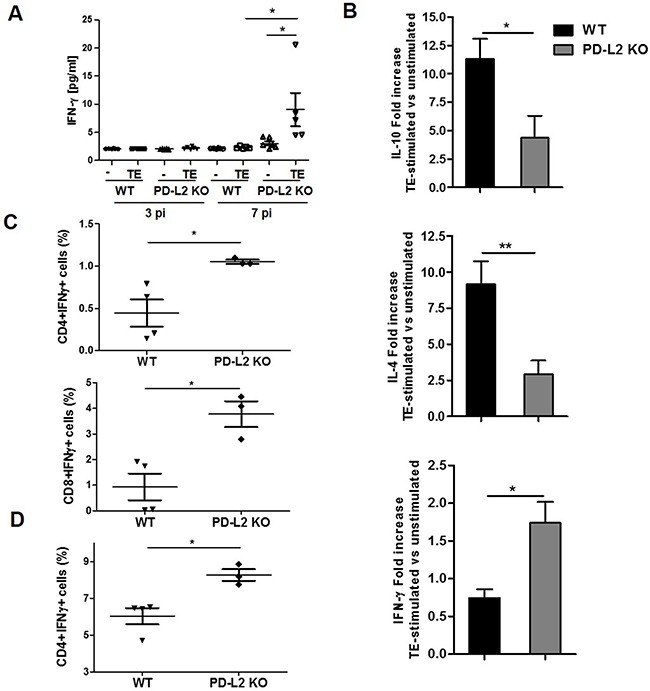
Th1 and Th2 cytokine levels in WT and PD-L2 KO infected mice Spleen mononuclear cells were obtained from WT and PD-L2 KO mice orally infected with 8 *F. hepatica* metacercariae. Then, the cells were stimulated with 80 μg/ml of TE or left unstimulated, and the cytokines measured in culture supernatants by ELISA. **A.** IFN-γ production by splenocytes at days 3 and 7 p.i. Dots represent mean cytokine levels of individual animals, lines represent the mean value *p<0.05 **B.** IL-10, IL-4 and IFN-γ production from splenocytes at day 21 p.i. Bars show fold increase of TE stimulated vs unstimulated cells. Data are expressed as mean ± SD *p<0.05;**p<0.005. **C.** CD4+ and CD8+ splenocytes producing IFN-γ are shown, each dot represents an individual animal *p<0.05. **D.** CD4+ intrahepatic lymphocytes producing IFN-γ are shown, with each dot representing an individual mouse * *p*<0.05. One representative experiment is depicted out of two.

Considering that PD-L2 is involved in both the regulation of T cell activation and tolerance [16], together with the fact that in our study *F. hepatica* infected PD-L2 KO mice showed a strong inflammatory liver damage, we hypothesized that PD-L2 expression is important for maintaining a survivable Th1 and Th2 cell balance during this infection. Thus, we evaluated cytokine production by splenocytes from WT and PD-L2 KO infected mice after being re-stimulated with *F. hepatica* TE for 72 h. Although at 7 days p.i. the Th2-type response was undetectable in both groups of mice (data not shown), we found low but significant levels of IFN-γ secreted by TE stimulated splenocytes from PD-L2 KO but not WT mice (Figure 3A). Subsequently, at day 21 p.i, TE stimulated splenocytes from PD-L2 KO mice showed a reduced ability to secrete IL-10 and IL-4 cytokines, while displaying an increased capacity to produce IFN-γ compared to splenocytes from WT mice (Figure 3B). Accordingly, the percentage of IFN-γ-producing-CD4+ and CD8+ splenocytes (Figure 3C), as well as IFN-γ-producing-CD4+ intrahepatic T cells were significantly increased in PD-L2 KO compared with WT mice (Figure 3D).

**Figure 4:**
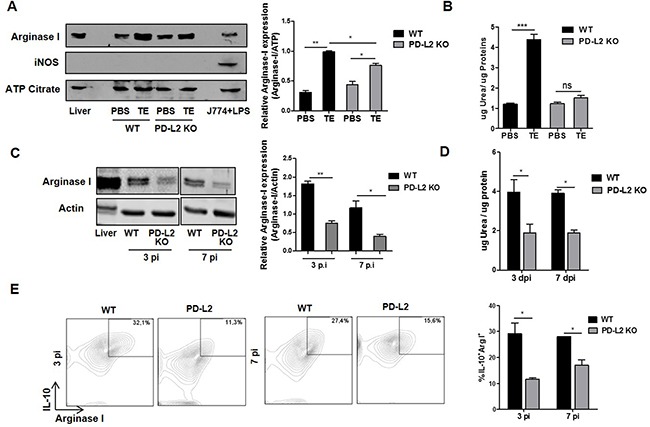
Reduced alternative activation of macrophages in total extract antigen (TE) injected or infected PD-L2 KO **A.** BALB/c WT and PD-L2 KO mice were injected with 80 μg of TE or PBS as control, and 24 h later arginase I and iNOS expression mice were analyzed in PC by Western blot. Mouse liver lysate was used as a positive control for arginase I expression and LPS-stimulated J774 cells were used as a positive control for iNOS expression. ATP Citrate expression was used as control loading. Right panel shows the densitometric analysis using GelPro software, *n* =2 experiments **p=0.0011; *p< 0.05. **B.** Arginase activity was evaluated by measuring urea levels in lysated PC, ***p=0.0003. **C.** WT and PD-L2 KO mice were infected with 8 *F. hepatica* metacercariae, and the PC were isolated at days 3 and 7 pi. Arginase I expression was measured by Western blot. Mouse liver lysate was used as a positive control for Arginase I expression, and Actin expression was analyzed as control loading. Right panel shows the densitometric analysis using GelPro software, *n* =2 experiments **p=0.0046; *p<0.05. **D.** Arginase activity was evaluated through urea production *p<0.05. **E.** PC were stained with anti-F4/80 antibody. Then, PC were fixed and permeabilized to evaluate arginase I and IL-10 expression by using APC labeled anti-arginase I antibody and a PE-Cy7 labeled anti-IL-10 antibody. Left panel: representative dot plots for each group of mice at days 3 and 7 p.i. are shown. Right panel: Bars display the percentage of IL-10+ arginase I+ cells on F4/80+ gated populations, *p <0.05. All experiments were repeated twice with similar results being obtained and are expressed as mean ± SD.

It has been previously demonstrated that *F. hepatica* infection as well as ES released by the parasite induce AAM, which contributes to suppressing the Th1-type immune response and promoting Th2 development [9, 19]. In addition, it has been shown that PD-L2 expression in *F. hepatica* ES treated-macrophages is important in suppressing and inducing the secretion of IFN-γ and IL-10 by T cells, respectively [19]. In order to investigate whether PD-L2 expression participates in the alternative activation of macrophages, TE was injected into WT and PD-L2 KO mice, and arginase I expression and activity were evaluated in peritoneal cells (PC) after 24 h. Although TE induced arginase I expression in PC from both groups of mice, PC from WT mice showed more than a two-fold increase in arginase I expression, in comparison to a minor increase observed in PC from PD-L2 mice (Figure 4A). In agreement, TE induced arginase I activity on PC from WT mice (Figure 4B).

Next, we tried to determine whether PD-L2 expression played a role in inducing the alternative activation of macrophages during *F. hepatica* infection, by assessing, arginase I expression and activity and the levels of arginase I/IL-10 expressing cells in PC from WT and PD-L2 KO infected mice. Remarkably, after 3 and 7 days p.i, PC from PD-L2 KO mice showed a significant reduction in arginase I expression and activity (Figure 4C and 4D) and in the frequency of arginase I+ IL-10+ gated on F4/80+ cells (Figure 4E), compared to WT mice.

In addition, as previously demonstrated during *F. hepatica* infection [8] and other helminth infections [18], the expression of iNOS, a marker of classical activation of macrophages, was not detected in PC from WT or PD-L2 KO TE-injected mice (Figure 4A).

Taken together, these results indicate that PD-L2 expression in macrophages is critical for the induction of AAM and for maintaining an optimal balance between the Th1- and Th2-type immune responses that assure host survival during *F. hepatica* infection.

## DISCUSSION

It is known that *F. hepatica* infection leads to a polarization of the immune response towards a Th2 profile in various animals, including sheep, cattle and mice [1]. In addition, the ability of the parasite to limit a pro-inflammatory Th1-type profile by promoting anti-inflammatory responses that depend on the Th2 response and Treg induction has also been demonstrated [4, 11, 12]. Nevertheless, it is still not fully understood how parasite antigens modulate antigen presenting cells (APC) activation to promote a Th2 response and control the Th1 immune response.

In this study, we showed that *F. hepatica* infection or intraperitoneal TE injection increased PD-L2 expression in macrophages from susceptible BALB/c mice. Concomitantly, we demonstrated that in the absence of PD-L2, *F. hepatica* infection induced fatal inflammatory liver damage, with high IFN-γ but reduced IL-10 and IL-4 production by splenocytes from infected mice. Based on these findings, we postulate that increased PD-L2 expression on macrophages at early p.i times (from day 1 to day 7) could promote control of the incipient Th1 response. Supporting this idea, Guasconi et al. [19] demonstrated using *in vitro* experimental settings that the blocking of PD-L2 in co-cultures of *F. hepatica*-ES-treated macrophages with anti-CD3-stimulated CD4+ T cells increases IFN-γ and decreases IL-10 production, without modifying IL-13 production. Similar results were observed by blocking PD-L2 in the co-cultures of bone marrow-derived dendritic cells (DC) stimulated with *Schistosoma mansoni* soluble egg antigens (ESA) and CD4+ T cells, where a significant rise in IFN-γ was observed [22].

The role of PD-L2 has been previously demonstrated in the control of the expansion of Th2 response [23]. Thus, we have previously demonstrated contrasting results for the PD-1-PD-L2 signaling pathway in peritoneal macrophages during *T. cruzi* infection, with PD-L1 but not PD-L2 expression involved in T cell suppression, while PD-L2 expression was involved in the control of arginase I and up-regulation of iNOS activity [24]. In addition, during *Nippostrongylus brasiliensis* infection, AAM expressing PD-L2 participate in the inhibition of Th2-type response, with *in vivo* blockade of PD-L2 inducing an enhancement of the Th2-type immune response in the lung [21]. Furthermore, it has also been demonstrated that during chronic filarial infection with the nematode *Litomosoides sigmodontis*, the diminished ability of Th2 cells to proliferate and produce IL-4, IL-5 and IL-2 cytokines is reversed by PD-L2 blockage, thereby confirming the role of PD-L2 in the control of the Th2 response [23]. Consequently, it is not clear why the PD-1-PD-L2 signaling pathway can alternatively control Th1 or Th2 responses in different experimental settings. Related to this, the role of AAM (which have a high expression of PD-L2) in regulating the immune response during helminth infection, is still not completely understood.

Although during helminth infections there is a predominance of the Th2 response, in some instances, such as the infection with the intestinal nematode *Trichuris muris* or the trematode *Schistosoma mansoni*, there is a strong underlying Th1 inflammatory response which may became pathogenic if the dominant Th2 response is inhibited. Thus, it is possible that depending on the microenvironment where the helminth parasite develops its life cycle, AAM might exert a control on excessive inflammatory Th1 or Th2 responses, otherwise this could result in being deleterious to the parasite and/or to the host [25].

Is not clear yet how PD-L2 signaling can contribute to controlling the Th1 response during early time of *F. hepatica* infection. We hypothesize that this could be due to a direct effect on Th1 cell proliferation or indirectly through the promotion of AAM, favoring the Th2 response, which is mainly involved in the control of the Th1 response. In this regard, our results together with previous reports [19] suggest that Dectin-1 signaling by *F. hepatica* products induces PD-L2 expression in peritoneal macrophages, with its expression being critical for inducing up-regulation of arginase I expression and activity on PC from both *F. hepatica* infected and TE injected mice, thus indicating a role for PD-L2 in the induction of AAM. Therefore, AMM promotes a Th2-type immune response that can control the Th1-type response by several mechanisms, including the secretion of IL-10, which inhibit the production of IL-12 by DC [26], or galectin-1, an immunomodulatory lectin that selectively induces Th1 apoptosis and promotes the Th2 function [27]. The control of Th1 cells during *F. hepatica* infection through the PD-L2 expression may be also attributable to Treg induction and/or expansion. However, the similar levels of CD4+ CD25+ Foxp3+ Treg cells in liver and spleen from WT and PD-L2 KO infected mice (data not shown), indicated the lack of involvement of Treg cells in this phenomenon.

During acute phase of *F. hepatica* infection, larva migration, hemorrhages, infiltration with eosinophils, lymphocytes, and macrophages, fibrosis, and necrotic areas have been reported [3]. Here, we have reported exacerbated liver damage in PD-L2 KO mice as denoted by hepatocyte enlargement, cytoplasmic vacuolization, and regions of cellular infiltration and foci of necrosis, hemorrhage and fibrosis. All these features might be associated with the unregulated Th1-type specific immune response developed by PD-L2 KO mice after *F. hepatica* infection. Consistent with these data, it has been reported that during HBV infection, the virus-specific Th1-type response is critical in the pathogenesis of fulminant hepatitis [28]. It has been demonstrated that during *T. cruzi* infection pro-inflammatory Th1-type cytokines as TNF can exert deleterious effects on tissues [29], however we found similar levels of TNF in sera from PD-L2 KO and WT mice (data not shown).

Here we showed that despite the increased recruitment of granulocytes and higher percentage of IFN-γ-producing CD4+ T cells in the livers of PD-L2 KO mice, the number of worms was similar to that observed in WT mice. This might be explained by the excessive production of NO by intrahepatic leukocytes, which in some infections is clearly associated with a more severe or even fatal disease outcome with possible underlying mechanisms including NO-mediated cytotoxicity and tissue damage, inhibition of T cell proliferation and/or induction of T cell apoptosis [30-32]. In agreement, during a thioacetamide-induced rat model of acute hepatic failure, the inhibition of iNOS activity by using the L-NAME and aminoguanidine significantly reduces the severity of damage and decreases mortality [33]. Moreover, increased NO, O_2_^−^ and ONOO production has been shown in IL-4 KO mice infected with *Schistosoma mansoni*, which correlated with greater liver damage and an inability to control the parasite burden in the liver [34].

Interestingly, according to our data, besides being relevant to the T cell polarization, during a helminth infection PD-L2 signaling seems to have a role in alternative macrophage polarization, suggesting a reverse or back-signaling through this molecule in macrophages. PD-L2 expression would allow macrophages to reach a phenotype that includes the expression and activity of arginase and the production of IL-10, two markers of AAM. Thus, in the absence of PD-L2, macrophages obtained at early times post *F. hepatica* infection, show an inability to be completely polarized to AAM compared with those from WT mice. These results are in contrast with those published by Kuipers et al, who showed that blocking of PD-L1 and PD-L2 with soluble PD-1-Ig fusion protein leads to a reduced DC maturation with an increased IL-10 production [35]. The basis of these differences are not clear yet, but we speculate that the use of soluble PD-1-Ig fusion protein can block both PD-L1 and PD-L2 molecules, while in our study we used macrophages from mice deficient only in PD-L2. Studies tending to identify differentially regulated genes in the absence of PD-L2 in infected macrophages, would allow to dissect the reverse signals that are activated through PD-L2 during a helminth infection.

Taken together, our data demonstrates that during *F. hepatica* infection, the PD-L2 signaling pathway is involved in alternative macrophage polarization as well as in the control of the underlying Th1-type immune response, and concomitantly plays a key role in determining the susceptibility to this heminth infection.

## MATERIALS AND METHODS

### Mice, antigen preparation and *F. hepatica* infection

BALB/c mice were obtained from the Comisión Nacional de Energía Atómica (CNEA; Buenos Aires, Argentina), and PD-L2 KO mice were generously donated by Dr Frank Housseau and Dr Drew Pardoll (Johns Hopkins University, Baltimore, MD). All mice were inbred and housed according to institutional guidelines, and used when between 2 and 3 months of age. The animal experiments were examined by The Institutional Experimentation Animal Committee (authorization no. 15-01-44195), which approved the animal handling and experimental procedures.

The total extract (TE) of *F. hepatica* was obtained from mature flukes of infected bovine livers, as previously described [36]. Mice were injected intraperitoneally with 80 μg of TE or PBS as control, and *F. hepatica* infection was carried out by giving 8 metacercariae orally (Baldwin Aquatics) per animal. At different time points post infection (p.i), mice were necropsied, and their livers, spleens, and PC were removed.

### PC harvesting

Resident PC from injected, infected or control animals were obtained by washing the peritoneal cavity with completed RPMI-1640 supplemented with 10% fetal bovine serum (FBS, PAA laboratories), L-glutamine (2 mM) and gentamicin (40 g/ml) and the cells were cultured in completed RPMI-1640. The PC were removed 1, 3 or 7 days p.i or 24 h post-injection from peritoneal cavity of WT or PD-L2 KO mice. Additionally, PC were collected from uninfected or PBS-injected animals and used as a control group. Cells were used to assay the surface expression of PD-L2, arginase expression and activity and IL-10 expression.

### Isolation of mouse intrahepatic leukocytes

Intrahepatic leukocytes (IHLs) were isolated from all mouse groups as previously described [37]. Briefly, liver in RMPI medium containing gentamicin (both from Gibco) plus 1% of FBS (PAA laboratories) was passed through 100-mm nylon meshes, red blood cells were removed using lysis buffer (Sigma) and finally IHLs were obtained after 20 min centrifugation (600 × *g*) in 35% and 70% bilayer Percoll (Sigma) gradients. The viable cell number was obtained by Trypan blue exclusion. Then, IHL were incubated with a probe and stained with anti-Ly6G, and cells DAF-FMDA probe for 15 min to determine Nitric oxide (NO) production by flow cytometry.

### Tissue preparation and staining

Livers were isolated from infected WT and PD-L2 KO mice fixed in 4% paraformaldehyde-PBS pH 7.4. Paraffin sections were cut and slides were stained with hematoxylin and eosin (H&E) and the histological features were scored. At least 5 livers were included in each experimental group. Images were obtained using a Nikon Eclipse TE 2000U equipped with a digital video camera.

### Fluorescence-Activated Cell-Scanning (FACS) Analysis

To examine PD-L2 expression, the PC were removed 1, 3 or 7 days p.i or 24 h post-injection from the peritoneal cavity of WT or PD-L2 KO mice. Additionally, PC were collected from uninfected animals or PBS injected animals and used as control groups. Cells were washed with saline solution 2% FBS and incubated with anti-mouse CD32/CD16 antibody for 20 minutes at 4°C to block Fc receptors. Then, cells were incubated with FITC labeled mAb against mouse F4/80 (BD Pharmingen) and with PE labeled mAb against mouse PD-L2 (BD Pharmingen) for 20 min at 4°C.

In addition intracellular expression of IFN-γ in spleen cells or IHLs was measured after treatment with 1 μg/ml brefeldin A (BD Pharmingen) and Phorbol myristate acetate (PMA) (Sigma) for 4 h at 37°C and 5% CO_2_. Spleen cells and IHLs were first stained with PerCP-conjugated anti-CD3, PE-conjugated anti-CD8 and APC-Cy7-conjugated anti-CD4 antibodies (BD Pharmingen). Once stained, the cells were permeabilized and fixed, and APC-conjugated anti-IFN-γ (BD Pharmingen) was added.

For the assessment of intracellular arginase I and IL10 expression, monensin (BD Pharmingen) was added to PC cultures at a final concentration of 2 μM during 4 h. Then, cells were stained with PE-F4/80 antibody, and then fixed and permeabilized with Citofix/Citoperm (BD Biosciences) followed by reacting with APC-labeled anti-arginase I antibody (R&D) and PE-Cy7-labeled anti-IL-10 antibody (Biolegend) for 45 min. Finally, cells were washed twice with saline solution 2% FBS, and stored at 4°C in the dark until being analyzed using a FACS flow cytometer (FACS Canto II, BD Biosciences).

Isotype controls were run with each set of samples and were used to define negative and positive cell populations. The results were processed using Flow Jo software.

### Arginase activity determination

PC (1×10^5^) were obtained from TE and PBS injected animals or from control and infected animals and the arginase activity was measured in cell lysates as described elsewhere [38, 39]. Briefly, cells were lysed with 50 μl of 0.1% Triton X-100 containing protease inhibitors and the mixture was stirred for 30 min and then 50 μl of 10 mM MnCl_2_ with 50 mM Tris-HCl were added to activate the enzyme for 10 min at 56 °C. Arginine hydrolysis was initiated by the addition of 25 μl of 0.5 M L-arginine, pH 9.7 at 37 °C for 45 min. The reaction was stopped using a mixture of acids, and the urea concentration was measured at 540 nm after the addition of 25 μl of α-isonitrosopropiophenone (dissolved in 100% ethanol), which was followed by heating at 95 °C for 45 min. These results are expressed as μg of urea per μg of protein.

### Cytokine determination

Cytokines were measured in culture supernatants using capture enzyme-linked immunosorbent assay (ELISA). IFN-γ (eBioscience), IL-10 and IL-4 (Biolegend) were used as paired monoclonal antibodies in combination with recombinant cytokine standards. Assays were performed according to the manufacturer's guidelines.

### Immunoblot analysis

To examine arginase I or iNOS expression, PC were removed from BALB/c or PD-L2 KO, TE or PBS injected mice. Additionally PC were isolated from BALB/c or PD-L2 KO infected mice. Cells were washed and lysed for 30 min at 4°C in RIPA buffer [1% Triton X-100 (v/v), 0.5% sodium deoxicolate (p/v), 0.1% sodium dodecyl sulfate (SDS)] containing protease inhibitor cocktail (Roche) and cell debris was spun down at 13,000 g for 15 min. Precipitates were removed, and aliquots of the cell lysates were diluted in SDS sample buffer, boiled at 100°C for 3 min, spun down, and applied to precast 10% acrylamide Tris-glycine gels at 40 μg protein/lane and run at 150 V for 1 h. Samples were transferred to nitrocellulose membrane (BioRad) at 100 V for 1 h and membranes were probed using rabbit anti-mouse arginase I polyclonal antibody or rabbit anti-mouse iNOS polyclonal antibody (Santa Cruz Biotechnology, Santa Cruz, CA) followed by peroxidase conjugated anti-rabbit antibody (Sigma Chemical Co.) and visualized using enhanced chemiluminescence (Pierce) for detection. Moreover rabbit anti-mouse ATP citrate or monoclonal anti-mouse β-actin antibodies (Cell Signalling Technology) were used for control loading.

### Statistical analysis

Statistical analyses were performed using the student's t-test and survival data were analyzed by the Gehan–Breslow Wilcoxon test. For both tests, p < 0.05 values were considered to be statistically significant.
